# A post hoc analysis of dalteparin versus oral anticoagulant (VKA) therapy for the prevention of recurrent venous thromboembolism (rVTE) in patients with cancer and renal impairment

**DOI:** 10.1007/s11239-016-1386-8

**Published:** 2016-06-25

**Authors:** Seth Woodruff, Guillaume Feugère, Paula Abreu, Joseph Heissler, Marcia T. Ruiz, Frank Jen

**Affiliations:** 1 Pfizer Inc, New York, NY USA; 2 Pfizer Inc, Kirkland, QC Canada

**Keywords:** Dalteparin, Low-molecular-weight heparin, Renal impairment, Thromboembolism, Vitamin K antagonist

## Abstract

Venous thromboembolism (VTE) is a common and serious complication in patients with cancer; treatment guidelines recommend extended therapy of ≥6 months with low-molecular-weight heparin (LMWH) for treatment and prevention of recurrent VTE (rVTE) in this population. This post hoc analysis used data from the CLOT study—a phase III, randomized, open-label, controlled study (N = 676)—to compare the efficacy and safety of dalteparin, a LMWH, versus vitamin K antagonist (VKA) for prevention of rVTE in patients with cancer and renal impairment (creatinine clearance <60 ml/min). Overall, 162/676 (24 %) patients had renal impairment at baseline. Patients received subcutaneous dalteparin 200 IU/kg once daily during month 1, followed by 150 IU/kg once daily for months 2–6; or VKA once daily for 6 months, with initial overlapping subcutaneous dalteparin 200 IU/kg once daily for ≥5 days until international normalized ratio was 2.0–3.0 for 2 consecutive days. Endpoints included the rates of rVTE (primary) and bleeding events. Overall, fewer dalteparin-treated patients (2/74 [2.7 %]) experienced ≥1 adjudicated symptomatic rVTE compared with VKA-treated patients (15/88 [17.0 %]; hazard ratio = 0.15 [95 % confidence interval 0.03–0.65]; *p* = 0.01). Bleeding event rates for both treatments were similar (*p* = 0.47). In summary, compared with VKA, dalteparin significantly reduced risk of rVTE in patients with cancer and renal impairment (*p* = 0.01) while exhibiting a comparable safety profile. This analysis supports dosing patients with renal impairment in accordance with patients with normal renal function; however, anti-Xa monitoring could be considered to further support safety in selected patients, particularly those with very severe renal impairment.

## Introduction

Patients with cancer experience a higher incidence of venous thromboembolism (VTE; acute deep vein thrombosis [DVT] and/or pulmonary embolism [PE]) than those without cancer, ranging from 3.8–30.7 % [1], depending on the cancer site [2–4], stage and grade [5, 6]. The risk of VTE is partly attributable to the hypercoagulable state induced by the cancer itself [4], and also can be significantly increased by use of cancer interventions such as chemotherapy, surgery, radiotherapy, hormonal therapy and other targeted therapies [4, 7–9]. Therefore, patients with cancer exhibit up to a sixfold higher risk of VTE than those without cancer, particularly in patients with advanced disease, hematological malignancies and certain types of solid tumors, e.g. lung, brain and gastrointestinal tract [4]. Risk of VTE is highest following the cancer diagnosis and when distant metastasis has occurred [3]. Development of VTE in patients with cancer is associated with poor prognosis and decreased survival [4, 10].

Standard treatment for VTE in patients with cancer is long-term anticoagulant therapy [9, 11, 12]. Previously, this included initial intravenous unfractionated heparin (UFH) or initial subcutaneous high-dose low-molecular-weight heparin (LMWH) overlapped and followed by an oral vitamin K antagonist (VKA) administered for >3 months [13]. However, dosing of VKA therapy requires inconvenient, close laboratory monitoring [14], and VTE recurrence rates in patients with cancer receiving this treatment regimen are higher than in patients without cancer [15]. Use of VKAs to treat VTE in patients with cancer also has proved challenging because of patient nausea, vomiting and anorexia, drug–drug interactions, poor venous access, bleeding complications and difficulty in maintaining the international normalized ratio (INR) within the targeted therapeutic range [9].

Because of the insufficiencies associated with VKA treatment, the CLOT study compared 6-month treatment with dalteparin, a unique LMWH with mean molecular weight of 6000 daltons, with initial dalteparin overlapped with and followed by 6 months of VKA for both the acute treatment and secondary prophylaxis of VTE in patients diagnosed with cancer and symptomatic proximal DVT and/or PE [16]. In summary, the results of the study showed a 52 % relative risk reduction of VTE recurrence over 6 months in the dalteparin-only arm compared with the VKA arm (*p* = 0.002); no significant differences were observed between groups in the incidence of major or minor bleeding events. Furthermore, the risk ratio of dalteparin to VKA for recurrent VTE (rVTE) remained statistically significant in favor of dalteparin when the model was adjusted for other factors found to be prognostic for VTE outcome (including extent and type of tumor). Since the publication of the CLOT study results, international guidelines have recommended long-term treatment with high-dose LMWH (≥6 months) as standard care for the acute treatment and secondary prophylaxis of VTE in patients with cancer [9, 11, 17].

Many patients with cancer also suffer from renal impairment, which is clinically relevant because reduced renal function can cause abnormalities in hemostasis, thereby increasing the patient’s prothrombotic tendency and bleeding risk [18]. In a French observational study of 4684 patients with varying types of cancer, a majority (57.4 %) of patients had abnormal creatinine clearance (CrCl; defined as <90 ml/min), of which 37.6, 18.5 and 1.3 % had a CrCl of 60–89, 30–59 or <30 ml/min, respectively [19]. Of note, in clinical practice, the frequency of renal impairment in patients with cancer can be underestimated if the diagnosis is based on serum creatinine (SrCr) levels [19]. An observational study of patients with cancer carried out at two French institutions reported that while 29/316 (9.2 %) patients showed elevated SrCr levels [20], 23 % of patients with normal SrCr (<110 µmol/l) had a CrCl of <80 ml/min, with evidence of impaired renal function. In addition to baseline renal impairment, cancer treatment itself can lead to, or worsen, renal impairment because such therapies can be nephrotoxic, particularly when used sequentially or in combination [21].

Reduced renal function also can impact the clinical outcomes of patients treated with anticoagulants because renal impairment can limit the elimination of these agents, potentially leading to bioaccumulation, and therefore, to adverse bleeding events. Of note, because of different pharmacokinetic profiles, the risk of bioaccumulation differs between classes of agents and between agents within the same class (e.g. LMWHs) [22]. For example, while UFH is cleared in a dose-dependent manner by the hepatic reticuloendothelial system, LMWHs primarily undergo renal elimination [23]. As a result, depending on dose and duration of treatment, LMWHs, as a class, which include bemiparin, dalteparin, danaparoid, enoxaparin, nadroparin and tinzaparin, can accumulate in patients with reduced renal function more than UFH [14, 22, 23]. However, LMWHs with higher mean molecular weights (e.g. dalteparin or tinzaparin—which was removed from the US market in 2011) undergo less renal (and more hepatic) elimination than LMWHs with lower mean molecular weights (e.g. bemiparin, enoxaparin or nadroparin). As a result, the risk of bioaccumulation of dalteparin or tinzaparin in patients with renal impairment is lower than that of LMWHs with lower mean molecular weights [24, 25].

Standard treatment for VTE in patients with cancer is long-term therapy with a LMWH. However, many of these patients have, or will develop, renal impairment, thereby increasing the risk of anticoagulant bioaccumulation that could lead to life-threatening adverse bleeding events. Because the risk of bioaccumulation owing to renal impairment differs significantly between LMWH agents, there is a critical need to have prospective published evidence on long-term use of specific LMWHs in patients with cancer, VTE and renal impairment to help guide treatment choices. The current analysis aims to address that gap for dalteparin. The exclusion criterion for the CLOT trial related to renal function was SrCr level >3× the upper limit of normal (ULN; 3.6 mg/dl). Consequently, a significant number of patients with some degree of renal impairment, as defined by CrCl, were enrolled in the study. In this post hoc subanalysis of CLOT, we evaluated the efficacy and safety of long-term high-dose dalteparin (therapeutic doses of 150–200 IU/kg/d as opposed to low prophylactic doses of 2500–5000 IU/d used for primary VTE prophylaxis) versus VKA in patients with cancer, VTE and normal/mild (CrCl ≥ 60 ml/min), moderate (30 ≤ CrCl < 60 ml/min) or severe (CrCl < 30 ml/min) renal impairment at baseline.

## Methods

### Study design and population

CLOT was an international, multicenter, open-label, randomized clinical trial of 676 patients presenting with cancer and VTE. A detailed description of the study design, population, treatment regimens and outcome measures has previously been published [16].

Per protocol, patients underwent 6-month treatment with dalteparin alone, or initial dalteparin overlapped with and followed by a VKA (i.e. warfarin or acenocoumarol). Those patients randomly assigned to dalteparin received once-daily subcutaneous injections of dalteparin 200 IU/kg (maximum daily dose 18,000 IU) for 1 month, followed by injections of ~150 IU/kg for the remaining 5 months. Those in the VKA group received once-daily VKA for 6 months, with initial overlapping subcutaneous dalteparin 200 IU/kg once daily for ≥5 days until INR was 2.0–3.0 for 2 consecutive days. Thereafter, laboratory monitoring of the INR was performed at each clinical assessment, once every 2 weeks or more frequently when clinically indicated, to adjust the oral anticoagulant dose. The INR level in the oral anticoagulant group was measured frequently to enhance the likelihood that patients were adequately treated. Using linear interpolation over time, it was estimated that the INR was in the therapeutic range 46 % of the time, below the range 30 % of the time, and above the range 24 % of the time.

Dose modification, including temporary interruption of treatment, was permitted when clinically indicated (i.e. if patients experienced transient thrombocytopenia or significant renal impairment: defined as SrCr level >3× ULN). Full dose was then reinstated once it could be resumed safely. In patients treated with dalteparin who developed significant renal impairment, the treatment dose was adjusted to maintain an anti-Xa therapeutic level of 1 IU/ml (range 0.5–1.5 IU/ml). If the steady-state anti-Xa level, measured 4–6 h after the last dalteparin injection, was below or above the therapeutic range, the dalteparin dose was altered by switching to the next highest or lowest prefilled syringe formulation dose, respectively—dalteparin was supplied as 1 mL single-dose syringes containing 5000, 7500, 10,000, 12,500, 15,000 or 18,000 IU anti-factor Xa—and the anti-Xa measurement was repeated after 3–4 new doses. This dose adjustment was repeated until the target anti-Xa therapeutic level was achieved. For those patients developing significant renal impairment while receiving VKA, no dose adjustment was made. During scheduled clinical assessments at baseline, days 7–10 and months 1, 3 and 6, blood samples were taken for CrCl measurements, and used to assess changes in renal function status over the course of the study.

The present post hoc analysis divided patients enrolled in CLOT into subgroups of those with normal renal function (CrCl ≥ 60 ml/min), and those with renal impairment at baseline, (CrCl < 60 ml/min), calculated using the Cockcroft–Gault formula [26]. For this analysis, patients with renal impairment were further classified as having either moderate (30 ≤ CrCl < 60 ml/min) or severe renal impairment (CrCl < 30 ml/min). Patients with normal renal function at baseline who developed renal impairment during the course of CLOT were excluded from this analysis.

### Outcome measures

The primary efficacy outcome was the rate of rVTE (i.e. the first episode of objectively documented, symptomatic, recurrent DVT or PE) in the intention-to-treat population. Secondary safety outcomes included clinically overt bleeding (any and major) and death in the as-treated population. Diagnostic criteria for rVTE and bleeding events have been described in detail elsewhere [16]. Briefly, rVTE was defined by ultrasonography or venography outcomes, and bleeding event severity was determined by its association with death, the site at which it occurred, requirements for blood transfusion, and impact on hemoglobin level [16].

### Statistical analysis

Baseline demographic and clinical characteristics of patients with renal impairment at study entry were summarized in frequency tables, with descriptive statistics used for quantitative variables. VTE recurrence and bleeding events were summarized by both frequency and proportion. A two-sided log-rank test was used to compare treatment effects of dalteparin and VKA on the risk of VTE recurrence and bleeding events. Significance was set at the 5 % level, and hazard ratios (HRs) and 95 % confidence intervals (CIs) were provided. Cox proportional hazard regression models were used to assess treatment effects on events.

Descriptive statistics and graphics were used to summarize changes in CrCl from baseline to lowest level during treatment, as well as any change in dalteparin dosing.

## Results

### Study population

Baseline demographic and clinical characteristics of patients stratified by renal function and treatment group are shown in Table 1. Overall, 162/676 (24 %) of patients in CLOT had renal impairment at baseline (dalteparin arm, 74; VKA arm, 88). Most of these patients had moderate impairment (dalteparin, 65/74 [88 %]; VKA, 82/88 [93 %]); only a small number of patients had severe renal impairment (dalteparin, 9/74 [12 %]; VKA, 6/88 [7 %]). Patients with renal impairment tended to be older and female, and weighed less than those with normal renal function; however, differences in these variables were anticipated because in addition to SrCr, they were used to calculate CrCl and therefore to determine patient renal function. Patients with renal impairment were well matched for age, body weight, SrCr and CrCl between the two treatment arms. Most patients had an Eastern Cooperative Oncology Group status score of 1 or 2, with the distribution of status scores being comparable among the four subgroups. An additional 91 patients in CLOT developed renal impairment at some point during treatment but were not included in this analysis.

**Table 1:** Baseline characteristics of patients with renal impairment or normal renal function, by treatment

Characteristic	Renal impairment		Normal renal function	
	Dalteparin n = 74	VKA n = 88	Dalteparin n = 264	VKA n = 250
Median (range) age (years)	71.0 (31.7–84.6)	73.9 (38.6–89.3)	61.7 (22.0–80.6)	61.1 (27.9–86.1)
Age <65 years, no. (%)	25 (33.8)	20 (22.7)	157 (59.5)	162 (64.8)
Age ≥65 years, no. (%)	49 (66.2)	68 (77.3)	107 (40.5)	88 (35.2)
Median (range) weight (kg)	64.0 (39.0–105.0)	65.0 (40.0–104.0)	75.5 (41.0–132.0)	75.0 (45.0–128.0)
Female, no. (%)	48 (64.9)	47 (53.4)	131 (49.6)	122 (48.8)
Median (range) CrCl (ml/min) (no. of patients)				
Normal^a^ (CrCl ≥60)	NA	NA	90.4 (60.0–233.5) [245]	92.5 (60.2–202.7) [225]
Moderate impairment (30 ≤ CrCl < 60)	48.5 (31.1–59.5) [65]	47.8 (31.5–59.7) [82]	NA	NA
Severe impairment (CrCl < 30)	27.6 (22.2–29.4) [9]	26.5 (21.0–29.6) [6]	NA	NA
Median (range) SrCr (mg/dl) (no. of patients)				
Normal (SrCr ≤1.2)	1.0 (0.6–1.2) [35]	1.0 (0.7–1.2) [45]	0.8 (0.3–1.2) [233]	0.8 (0.4–1.2) [208]
High (SrCr > 1.2)	1.6 (1.2–3.3) [39]	1.5 (1.2–2.9) [43]	1.3 (1.2–1.4) [12]	1.4 (1.2–2.0) [17]

*VKA* vitamin K antagonist, *CrCl* creatinine clearance, *SrCr* serum creatinine, *NA* not applicable

^a^19 and 25 patients were missing CrCl baseline data in the dalteparin and VKA groups, respectively

### Dosing and treatment duration

**Fig. 1:**
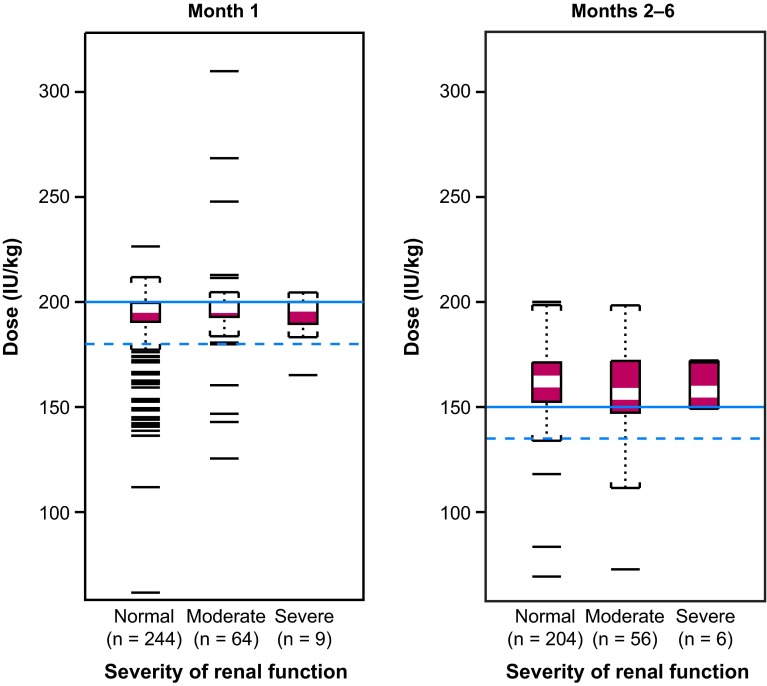
Summary of average dalteparin dose (IU/kg) during month 1 and months 2–6 of treatment. The *shaded box* at the center contains 50 % of the data; the *white bar* within indicates the median. The *solid horizontal lines* are drawn at the prescribed doses, i.e. 200 IU/kg for month 1 and 150 IU/kg for months 2–6 of the study. The *dotted lines* indicate values at ~90 % of the prescribed levels, respectively

A summary of the average dalteparin dose administered to patients in each of the three renal function subgroups during month 1 and months 2–6 is presented in Fig. 1. As shown, the distributions of the received dalteparin doses were comparable between renal function subgroups during months 2–6, with median doses near the dose levels prespecified in the protocol and no differences between subgroups. Irrespective of renal function at baseline, the majority (>84 %) of patients received dalteparin at ≥90 % of the prescribed levels. During month 1, the mean doses received by patients with normal renal function, moderate renal impairment and severe renal impairment were: 190.6, 196.0 and 193.3 IU/kg, respectively; during months 2–6, the mean doses were 160.3, 157.2 and 159.5 IU/kg, respectively. Each of these six mean doses was within the 5 % range of the dosages specified in the CLOT study treatment protocol.

Distribution of dalteparin doses seen in patients with renal impairment was similar to that for patients with normal renal function, i.e. there was no systematic reduction of dalteparin dosage in patients with renal impairment (including patients with severe impairment). Among the 74 dalteparin-treated patients with renal insufficiency at baseline, only 1 patient had a temporary dose reduction owing to increased anti-Xa levels. Similarly, of the 91/676 (13 %) patients in CLOT who developed renal impairment during the course of the study, 2/91 (2 %) had dose reductions owing to increased anti-Xa levels.

### VTE recurrence

**Fig. 2:**
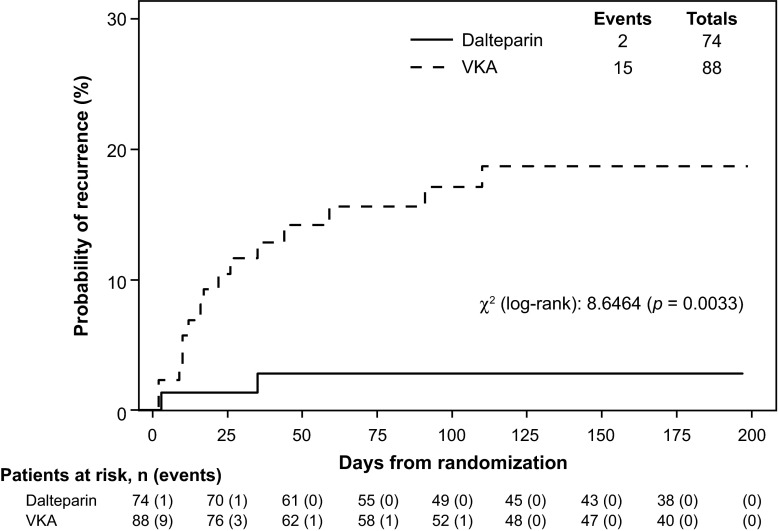
Time to first recurrent venous thromboembolism (deep vein thrombosis/pulmonary embolism) during the 6-month study period for patients with renal impairment. *p* value calculated using log-rank test. *VKA* vitamin K antagonist

Overall, 2/74 (2.7 %) dalteparin-treated patients with renal impairment (moderate impairment, 2) and 15/88 (17.0 %) VKA-treated patients with renal impairment (moderate impairment, 14; severe impairment, 1) in the intention-to-treat population, experienced ≥1 adjudicated symptomatic rVTE during the 6-month study period (cox proportional hazard model: HR [95 % CI], 0.15 [0.03–0.65] in favor of dalteparin; *p* = 0.01; Table 2). A Kaplan–Meier curve showing time to first rVTE during the 6-month study period for patients with renal impairment is presented in Fig. 2 (*p* value calculated using log-rank test).

**Table 2:** Comparison of treatment effects on first VTE recurrence, first any bleeding and first major bleeding in patients with renal impairment

Variable	Treatment	Patients at risk (no.)	Events	%	*p* value^a^	Hazard ratio (95 % CI)
VTE (n = 162)^b^	Dalteparin	74	2	2.7	0.0111	0.148 (0.034–0.647)
	VKA	88	15	17.0		
Any bleeding (n = 161)^c^	Dalteparin	74	15	20.3	0.4658	0.781 (0.402–1.517)
	VKA	87	21	24.1		
Major bleeding (n = 161)^c^	Dalteparin	74	7	9.5	0.6511	1.287 (0.432–3.834)
	VKA	87	6	6.9		

*VTE* venous thromboembolism, *CI* confidence interval, *VKA* vitamin K antagonist, *ITT* intention-to-treat, *AST* as-treated

^a^Cox proportional model with treatment as covariate

^b^ITT patients

^c^AST patients

Cox proportional hazard models were used to evaluate the potential influence of baseline renal function on the likelihood of VTE recurrence. Specifically, both numerical CrCl values and a derived indicator variable (based on a CrCl less than or greater than 60 ml/min) were used as renal function indices and as possible explanatory variables in two Cox models calculated with or without prognostic variables. Prognostic variables included extent of tumor (nonmetastatic vs. metastatic), type of tumor (gastrointestinal vs. breast, lung vs. breast, genitourinary vs. breast, hematological vs. breast, other vs. breast), current smoking status (smoker vs. nonsmoker) and age at study entry. Neither the numerical CrCl value nor the derived indicator value was found in either Cox model to influence the treatment effect of dalteparin versus VKA on VTE recurrence.

### Bleeding events

First instances of any bleeding or major bleeding were determined in the as-treated population according to treatment and renal function status (Tables 2, 3).

**Table 3:** VTE recurrence and bleeding events in the subgroups determined by treatment and renal function at baseline

Treatment	Renal function^a^	VTE		Any bleeding		Major bleeding	
		At risk^b^ (no.)	Events, no. (%)	At risk^c^ (no.)	Events, no. (%)	At risk^c^ (no.)	Events, no. (%)
Dalteparin	Missing baseline CrCl	19	3 (15.8)	19	2 (10.5)	19	2 (10.5)
	Normal	245	22 (9.0)	245	29 (11.8)	245	10 (4.1)
	Renal impairment	74	2 (2.7)	74	15 (20.3)	74	7 (9.5)
	Moderate impairment	65	2 (3.1)	65	10 (15.4)	65	5 (7.7)
	Severe impairment	9	0	9	5 (55.6)	9	2 (22.2)
VKA	Missing baseline CrCl	25	2 (8.0)	24	4 (16.7)	24	1 (4.2)
	Normal	225	36 (16.0)	224	37 (16.5)	224	5 (2.2)
	Renal impairment	88	15 (17.0)	87	21 (24.1)	87	6 (6.9)
	Moderate impairment	82	14 (17.1)	81	18 (22.2)	81	5 (6.2)
	Severe impairment	6	1 (16.7)	6	3 (50.0)	6	1 (16.7)

*VTE* venous thromboembolism, *CrCl* creatinine clearance, *VKA* vitamin K antagonist, *ITT* intention-to-treat, *AST* as-treated

^a^Normal: CrCl ≥ 60 ml/min; moderate impairment: 30 ≤ CrCl < 60 ml/min; severe impairment: CrCl < 30 ml/min

^b^ITT population

^c^AST population, 3 patients less

### Any bleeding

The proportion of patients with renal impairment at baseline in the as-treated population experiencing ≥1 bleeding episode was higher in the VKA treatment arm than in the dalteparin treatment arm (21/87 [24.1 %] vs. 15/74 [20.3 %]; Table 2); however, the between-group difference in cumulative probability of any bleeding event was not statistically significant (*p* = 0.47).

The rate of any bleeding increased as renal function declined in both treatment groups (Table 3). Among as-treated patients with moderate renal impairment, bleeding events were experienced by 10/65 (15.4 %) of those treated with dalteparin and 18/81 (22.2 %) of those treated with VKA. In patients with severe renal impairment, bleeding events were experienced by 5/9 (55.6 %) of those receiving dalteparin and 3/6 (50.0 %) of those receiving VKA.

### Major bleeding

Dalteparin was associated with a numerically higher incidence of ≥1 adjudicated major bleeding event compared with VKA, but the between-group difference in cumulative probability of such an event was not statistically significant (*p* = 0.65; Table 2). Specifically, incidence of a major bleeding event in as-treated patients with moderate renal impairment was 5/65 (7.7 %) in patients treated with dalteparin and 5/81 (6.2 %) in patients treated with VKA. Incidence of major bleeding events in as-treated patients with severe renal impairment was 2/9 (22.2 %) with dalteparin and 1/6 (16.7 %) with VKA (Table 3); there were few major bleeding events seen in patients with moderate or severe renal impairment in either treatment group.

### Death rates

The overall death rate among patients with renal impairment during the 6-month study period was 79/162 (48.8 %). The death rate in dalteparin-treated patients was 36/74 (48.6 %) and was 43/88 (48.9 %) in VKA-treated patients.

### Change in renal function status

Change in renal function status during treatment in the intention-to-treat population is summarized in Table 4. Results indicate that 79 % of patients treated with dalteparin and 75 % of patients treated with VKA who had normal renal function at baseline maintained this status during the treatment period, while 75 % of dalteparin-treated patients and 79 % of VKA-treated patients with moderate renal impairment at baseline either maintained or improved their renal function during the treatment period (Table 4).

**Table 4:** Frequency of renal function change from baseline to worst levels during treatment (ITT population)

Treatment	Baseline renal function^a^	Baseline (no.)	Worst renal function^a^ experienced during treatment, no. (%)			
			CrCl missing	Normal	Moderate impairment	Severe impairment
Dalteparin (n = 338)	CrCl missing	19	1 (5)	13 (68)	4 (21)	1 (5)
	Normal	245	10 (4)	193 (79)	40 (16)	2 (1)
	Moderate impairment	65	5 (8)	6 (9)	43 (66)	11 (17)
	Severe impairment	9	0	0	0	9 (100)
VKA (n = 338)	CrCl missing	25	2 (8)	16 (64)	5 (20)	2 (8)
	Normal	225	20 (9)	168 (75)	31 (14)	6 (3)
	Moderate impairment	82	6 (7)	11 (13)	54 (66)	11 (13)
	Severe impairment	6	0	0	1 (17)	5 (83)

*ITT* intention-to-treat, *CrCl* creatinine clearance, *VKA* vitamin K antagonist

^a^Normal: CrCl ≥ 60 ml/min; moderate impairment: 30 ≤ CrCl < 60 ml/min; severe impairment: CrCl < 30 ml/min

**Fig. 3:**
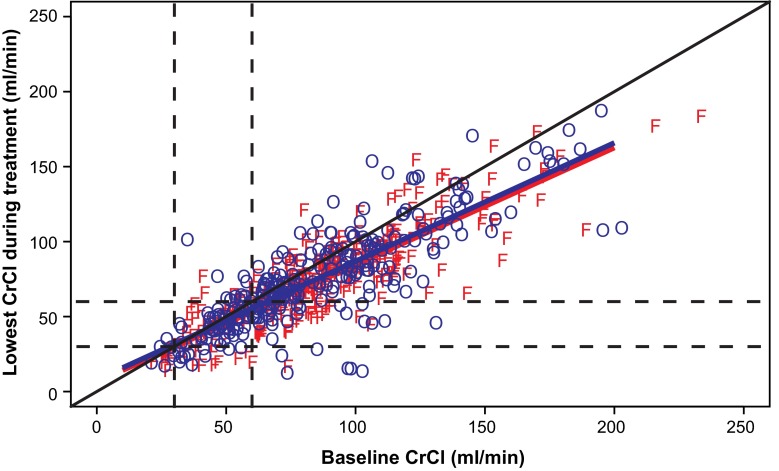
Lowest creatinine clearance (CrCl) during treatment versus baseline CrCl in patients given dalteparin (F) or vitamin K antagonist (VKA [O]; intention-to-treat population). The *solid black diagonal line* is the line of identity (y = x). Linear regression lines, i.e. the *solid blue diagonal line* for patients receiving VKA and the *solid red diagonal line* for those receiving dalteparin, have been added to indicate trends. The *black dashed lines* signify CrCl 30 ml/min and CrCl 60 ml/min

CrCl at baseline and at its lowest point during treatment is shown for the intention-to-treat population in Fig. 3. Most measured CrCl values were below the line of identity and the slopes of the regression lines were <1, indicating that most patients with or without renal impairment at baseline experienced a decrease in CrCl level during treatment. However, this change in CrCl was not sufficiently large enough to change the classification of renal function (e.g. normal, moderate impairment, severe impairment) assigned to most patients. The regression lines for dalteparin and VKA were similar, thereby indicating that change in renal function was comparable between treatment groups and that stability of renal function over the course of the study was similar.

## Discussion

In this post hoc analysis of prespecified endpoints, patients with cancer who had both acute VTE and impaired renal function at baseline demonstrated an 86.5 % relative risk reduction of developing rVTE when treated with dalteparin versus VKA. In the dalteparin arm, VTE recurrence event rates decreased as baseline renal function declined from normal to moderate or severe (although the number of patients in the severe group was small), while VTE recurrence event rates remained stable as renal function declined in the VKA arm. In both treatment groups, rates of any bleeding were higher in patients with renal impairment (moderate or severe) than in patients with normal renal function, respectively (20.3 and 11.8 % for dalteparin; 24.1 and 16.5 % for VKA).

Overall, the post hoc efficacy findings in patients with moderate and severe renal impairment were consistent with those described for the full CLOT study population where dalteparin reduced the cumulative risk of rVTE at 6 months by 52 % versus VKA, without increasing risk of bleeding [16]. In general, safety findings were similar between patients with renal impairment and those comprising the entire CLOT study population. Namely, there were no significant differences in rates of any bleeding event or major bleeding events between dalteparin and VKA treatment groups [16]. A higher death rate of 49 % was observed among patients with renal impairment, compared with 40 % among all patients in CLOT. Comparable safety findings were recently reported for dalteparin from the DALTECAN study, a single-arm 12-month safety study modeled after CLOT that also included patients with renal impairment [27].

Another large randomized trial modeled after CLOT but evaluating a different LMWH (tinzaparin) versus VKA, also was recently published [28]. In this study, known as CATCH, 900 patients with active cancer and VTE were randomized to tinzaparin 175 IU/kg once daily for 6 months or to tinzaparin 175 IU/kg once daily for 5–10 days followed by warfarin for 6 months [28]. Overall, in contrast to CLOT, the results reported no significant reduction in a composite measure of rVTE, overall mortality or major bleeding events following 6-month treatment with tinzaparin [28]. A subanalysis of CATCH directly investigated the impact of renal impairment on rVTE or clinically relevant bleeding (CRB) incidences; among 129 patients with CrCl < 60 ml/min, 14 % developed rVTE and 20 % had CRB. Of 733 patients with normal renal function, 8 % developed rVTE, while 15 % had CRB. For those patients with CrCl < 60 ml/min, no statistically significant differences were observed in rVTE and CRB incidences between the tinzaparin and warfarin treatment arms [29].

This post hoc analysis of CLOT and the subanalysis of CATCH are the earliest studies reporting long-term 6-month use of specific LMWHs to treat VTE in patients with active cancer and renal impairment. These two analyses provide evidence suggesting that both dalteparin and tinzaparin, although different agents, have safety profiles similar to VKA in this indication and patient cohort. In these two studies, bleeding events were markedly increased when anticoagulant treatment (LWMH or VKA) was administered to patients with renal impairment (compared with patients with normal renal function), but when LMWH treatment was compared with VKA, there was no evidence of excess bleeding (as would be expected should bioaccumulation be occurring). Intriguingly, this post hoc analysis of CLOT documented a larger and statistically significant reduction in rVTE with dalteparin in renally impaired patients than was previously demonstrated in the entire CLOT study population. The subanalysis of CATCH, however, reported similar rates of rVTE with tinzaparin and VKA in renally impaired patients, and these rates are almost twofold higher than observed in the entire CATCH study population. The difference in efficacy observed between dalteparin and tinzaparin versus VKA in this indication is unexplained and could be due to chance, or because of differences in pharmacodynamics that become clinically relevant in the hypercoagulable state induced by cancer, and/or because of study design variations.

In terms of dosing, the mean dalteparin dose received by patients within the three renal subgroups (normal, moderate impairment, severe impairment) fell within ±5 % of the range specified in the CLOT study protocol. Specifically, the protocol defined the month 1 dose to be 200 IU/kg per day, an amount designed to provide therapeutic levels of anticoagulation for acute VTE at a time when recurrence rates are highest in patients with cancer [15]. To reduce risk of bleeding complications during months 2–6, the dose was reduced to approximately 75–83 % of the initial daily dose (i.e. about 150 IU/kg per day), without regard to renal function and in the absence of serum anti-Xa monitoring. Dalteparin dose modification or interruption was, however, permitted if patients experienced transient thrombocytopenia or significant renal impairment. Importantly, we found no systematic or widespread reduction of dalteparin dosage in patients with renal impairment, even in those with CrCl < 30 ml/min. Indeed, elevated anti-Xa levels led to a reduction in the dose of dalteparin in only 1/74 patients enrolled with a baseline CrCl < 60 ml/min.

Of note, not every hospital or clinic has ready access to anti-Xa monitoring tests. Based on previously published data, current American Society of Clinical Oncology guidelines do advise that if anti-Xa monitoring is unavailable for patients with moderate to severe renal impairment, then UFH and VKAs are safer options for initial and long-term treatment, respectively [17]. Similarly, current National Comprehensive Cancer Network guidelines, which highlight that only limited data are available to support the clinical relevance of anti-Xa monitoring, recommend generally limiting the use of LMWHs in patients with renal insufficiency, rather than close monitoring [30].

Some limitations of this exploratory post hoc analysis are evident. First, patients with SrCr > 3.6 mg/dl were excluded from CLOT, thus limiting enrollment of patients with severe renal insufficiency. Indeed, only 15 patients enrolled in the two treatment arms had severe renal impairment (i.e. CrCl < 30 ml/min). However, the prevalence of severe renal insufficiency in patients with cancer has been shown to be low [19]. Second, CLOT did not stratify patients by the presence or severity of renal impairment, nor was it powered to detect between-treatment differences for most subgroups. However, a review of baseline characteristics of patients with CrCl < 60 ml/min demonstrated reasonable comparability between the dalteparin and VKA subgroups, thus permitting a statistical analysis of the treatment effects of dalteparin versus VKA on efficacy and safety endpoints in this subpopulation. Despite these limitations, a review of currently available published literature indicated that CLOT offered the largest safety and efficacy database of patients with cancer and renal impairment who were given long-term (>30 days) VKA and LMWH therapy.

We omitted 91 patients from our statistical analyses who developed renal impairment during the course of CLOT because heterogeneity in the cause, duration and course of renal function in patients who developed renal dysfunction during the study would have invalidated comparisons between patients with and without renal impairment. Those patients who transitioned from normal to impaired renal status were not a statistically well-defined subpopulation, i.e. their status was based on an outcome (change in renal function) that occurred at variable times during treatment. Moreover, the combined population of patients with renal impairment—those identified at baseline plus those who became impaired during treatment—was not truly randomized between treatment arms, further making any comparisons statistically invalid.

Efficacy and safety data from this post hoc analysis provide useful information to clinicians considering use of dalteparin to prevent rVTE in patients with cancer and renal impairment. However, the results cannot be extrapolated to other LMWHs because the pharmacokinetic and pharmacodynamic profiles of LMWHs clearly differ [31]. This reflects differences in manufacturing processes [32] and also mean molecular weight [24], which is considered to determine the extent to which LMWHs undergo renal elimination [25].

Given the findings of the current analysis, a pharmacoeconomic analysis is planned to test the hypothesis that dalteparin may be both cost effective and cost saving when used in patients with cancer and renal impairment. This analysis builds on a study by Dranitsaris et al. that used health care resource data collected during CLOT to conduct a patient-level economic analysis from a Canadian health care perspective. The investigators reported that secondary prophylaxis with dalteparin as an alternative to VKA in patients with cancer was economically attractive [33].

## Conclusions

In this post hoc analysis of patients with active cancer and acute VTE, high-dose long-term treatment with dalteparin significantly reduced the risk of rVTE in patients with renal impairment and had a comparable safety profile versus VKA. These findings, which are specific for dalteparin, are consistent with results reported previously from both the full CLOT study [16], which evaluated clinical outcomes with dalteparin and VKA in a large cohort of patients with active cancer and VTE who were treated for 6 months, and DALTECAN, a 12-month safety study [27].

No evidence emerged from this analysis to support dosing of dalteparin in patients with renal impairment other than with the dosages recommended in the CLOT study protocol. Baseline renal impairment leading to elevated anti-Xa levels was an exceptionally rare reason for dose reduction during dalteparin treatment. However, bleeding events were more common in the presence of renal insufficiency, particularly in patients with severe renal impairment. Therefore, in line with American College of Chest Physicians recommendations, clinicians who select dalteparin for the treatment of VTE in patients with cancer and renal impairment should continue to consider monitoring steady-state anti-Xa levels [22].

## References

[1] Prandoni P (2005). How I treat venous thromboembolism in patients with cancer. Blood.

[2] Deitcher SR (2003). Cancer-related deep venous thrombosis: clinical importance, treatment challenges, and management strategies. Semin Thromb Hemost.

[3] Blom JW, Doggen CJ, Osanto S, Rosendaal FR (2005). Malignancies, prothrombotic mutations, and the risk of venous thrombosis. JAMA.

[4] Heit JA (2005). Cancer and venous thromboembolism: scope of the problem. Cancer Control.

[5] Chew HK, Wun T, Harvey D, Zhou H, White RH (2006). Incidence of venous thromboembolism and its effect on survival among patients with common cancers. Arch Intern Med.

[6] Königsbrügge O, Pabinger I, Ay C (2014). Risk factors for venous thromboembolism in cancer: novel findings from the Vienna Cancer and Thrombosis Study (CATS). Thromb Res.

[7] Prandoni P, Falanga A, Piccioli A (2005). Cancer and venous thromboembolism. Lancet Oncol.

[8] Nalluri SR, Chu D, Keresztes R, Zhu X, Wu S (2008). Risk of venous thromboembolism with the angiogenesis inhibitor bevacizumab in cancer patients: a meta-analysis. JAMA.

[9] Mandala M, Falanga A, Roila F, Group EGW (2011). Management of venous thromboembolism (VTE) in cancer patients: ESMO Clinical Practice Guidelines. Ann Oncol.

[10] Sørensen HT, Mellemkjaer L, Olsen JH, Baron JA (2000). Prognosis of cancers associated with venous thromboembolism. N Engl J Med.

[11] Guyatt GH, Akl EA, Crowther M, Gutterman DD, Schuünemann HJ (2012). Executive summary: antithrombotic therapy and prevention of thrombosis, 9th ed: American College of Chest Physicians evidence-based clinical practice guidelines. Chest.

[12] McRae SJ, Ginsberg JS (2004). Initial treatment of venous thromboembolism. Circulation.

[13] Hyers TM, Agnelli G, Hull RD, Morris TA, Samama M, Tapson V, Weg JG (2001). Antithrombotic therapy for venous thromboembolic disease. Chest.

[14] Ansell J, Hirsh J, Hylek E, Jacobson A, Crowther M, Palareti G (2008). Pharmacology and management of the vitamin K antagonists: American College of Chest Physicians evidence-based clinical practice guidelines (8th Edition). Chest.

[15] Prandoni P, Lensing AW, Piccioli A, Bernardi E, Simioni P, Girolami B, Marchiori A, Sabbion P, Prins MH, Noventa F, Girolami A (2002). Recurrent venous thromboembolism and bleeding complications during anticoagulant treatment in patients with cancer and venous thrombosis. Blood.

[16] Lee AY, Levine MN, Baker RI, Bowden C, Kakkar AK, Prins M, Rickles FR, Julian JA, Haley S, Kovacs MJ, Gent M (2003). Low-molecular-weight heparin versus a coumarin for the prevention of recurrent venous thromboembolism in patients with cancer. N Engl J Med.

[17] Lyman GH, Khorana AA, Kuderer NM, Lee AY, Arcelus JI, Balaban EP, Clarke JM, Flowers CR, Francis CW, Gates LE, Kakkar AK, Key NS, Levine MN, Liebman HA, Tempero MA, Wong SL, Prestrud AA, Falanga A (2013). Venous thromboembolism prophylaxis and treatment in patients with cancer: American Society of Clinical Oncology clinical practice guideline update. J Clin Oncol.

[18] Jalal DI, Chonchol M, Targher G (2010). Disorders of hemostasis associated with chronic kidney disease. Semin Thromb Hemost.

[19] Launay-Vacher V, Oudard S, Janus N, Gligorov J, Pourrat X, Rixe O, Morere JF, Beuzeboc P, Deray G (2007). Prevalence of renal insufficiency in cancer patients and implications for anticancer drug management: the Renal Insufficiency and Anticancer Medications (IRMA) study. Cancer.

[20] Launay-Vacher V, Izzedine H, Rey JB, Rixe O, Chapalain S, Nourdine S, Paci A, Bourget P, Deray G (2004). Incidence of renal insufficiency in cancer patients and evaluation of information available on the use of anticancer drugs in renally impaired patients. Med Sci Monit.

[21] Launay-Vacher V, Aapro M, De Castro G, Cohen E, Deray G, Dooley M, Humphreys B, Lichtman S, Rey J, Scotté F, Wildiers H, Sprangers B (2015). Renal effects of molecular targeted therapies in oncology: a review by the Cancer and the Kidney International Network (C-KIN). Ann Oncol.

[22] Garcia DA, Baglin TP, Weitz JI, Samama MM (2012). Parenteral anticoagulants: antithrombotic therapy and prevention of thrombosis, 9th ed: American College of Chest Physicians evidence-based clinical practice guidelines. Chest.

[23] Hirsh J, Warkentin TE, Shaughnessy SG, Anand SS, Halperin JL, Raschke R, Granger C, Ohman EM, Dalen JE (2001). Heparin and low-molecular-weight heparin: mechanisms of action, pharmacokinetics, dosing, monitoring, efficacy, and safety. Chest.

[24] Nagge J, Crowther M, Hirsh J (2002). Is impaired renal function a contraindication to the use of low-molecular-weight heparin?. Arch Intern Med.

[25] Johansen KB, Balchen T (2013). Tinzaparin and other low-molecular-weight heparins: what is the evidence for differential dependence on renal clearance?. Exp Hematol Oncol.

[26] Cockcroft DW, Gault MH (1976). Prediction of creatinine clearance from serum creatinine. Nephron.

[27] Francis CW, Kessler CM, Goldhaber SZ, Kovacs MJ, Monreal M, Huisman MV, Bergqvist D, Turpie AG, Ortel TL, Spyropoulos AC, Pabinger I, Kakkar AK (2015). Treatment of venous thromboembolism in cancer patients with dalteparin for up to 12 months: the DALTECAN study. J Thromb Haemost.

[28] Lee AY, Kamphuisen PW, Meyer G, Bauersachs R, Janas MS, Jarner MF, Khorana AA (2015). Tinzaparin vs warfarin for treatment of acute venous thromboembolism in patients with active cancer: a randomized clinical trial. JAMA.

[29] Bauersachs R, Lee AYY, Kamphuisen P, Meyer G, Janas MS, Jarner MF, Khorana AA (2015). Long-term tinzaparin versus warfarin for treatment of venous thromboembolism (VTE) in cancer patients-analysis of renal impairment (RI) in the CATCH study [abstract AS214]. J Thromb Haemost.

[30] National Comprehensive Cancer Network Clinical Practice Guidelines in Oncology (2015) Cancer-associated venous thromboembolic disease. https://www.nccn.org/professionals/physician_gls/pdf/vte.pdf. Accessed 18 May 2016

[31] Fareed J, Adiguzel C, Thethi I (2011). Differentiation of parenteral anticoagulants in the prevention and treatment of venous thromboembolism. Thromb J.

[32] Linhardt RJ, Gunay NS (1999). Production and chemical processing of low molecular weight heparins. Semin Thromb Hemost.

[33] Dranitsaris G, Vincent M, Crowther M (2006). Dalteparin versus warfarin for the prevention of recurrent venous thromboembolic events in cancer patients: a pharmacoeconomic analysis. Pharmacoeconomics.

